# “A Father and Daughter Presentation of Autosomal Dominant Sleep‐Related Hypermotor Epilepsy Via a DEPDC5 Deletion in Exon 28”

**DOI:** 10.1155/crnm/5986538

**Published:** 2026-11-01

**Authors:** Julianna L. Barbaro, Elisabeth M. Benham, Ayushi Ambekar, Chetan S. Nayak

**Affiliations:** ^1^ Virginia Tech Carilion School of Medicine Roanoke Virginia USA vt.edu; ^2^ Fralin Biomedical Research Institute Roanoke Virginia USA; ^3^ Division of Neurology Carilion Clinic Roanoke Virginia USA carilionclinic.org

**Keywords:** autosomal dominant sleep-related hypermotor epilepsy, brivaracetam, DEPDC5, lacosamide

## Abstract

Sleep‐related hypermotor epilepsy (SHE) is a form of focal epilepsy that can be caused by a pathogenic autosomal‐dominant DEP domain containing Protein 5 (DEPDC5) variant. Given its variable clinical presentation and etiology, SHE can be challenging to diagnose and treat effectively. This case describes a father–daughter pair who experienced sleep‐related seizures with similar semiologies and were later diagnosed with autosomal dominant sleep‐related hypermotor epilepsy (ADSHE) after genetic test results revealed that they each had a DEPDC5 deletion in Exon 28. Currently, seizures are well controlled in the father with lacosamide plus brivaracetam and in the daughter with lacosamide alone. Due to the technically difficult nature inherent in diagnosing ADSHE, both father and daughter experienced diagnostic and treatment delays. This case report highlights the importance of early pattern recognition and genetic testing in patients with clinical features of ADSHE and expands upon the current understanding of clinical presentations of pathogenic DEPDC5 variants.

## 1. Introduction

Sleep‐related hypermotor epilepsy (SHE) is a focal epilepsy syndrome characterized by brief, abrupt onset, stereotypical, hypermotor episodes usually occurring during Stage II nonrapid eye movement sleep. The pathogenesis of ADSHE is complex and not fully understood but can be related to genetic variants with or without structural abnormalities [1].

SHE is rare with a prevalence rate of 1.8 per 100,000 people [1]. Genetic variants are the underlying etiology in 19% of familial SHE and 7% of sporadic cases [2]. There are three categories of genes associated with SHE: (1) neuronal ligand and ion gated channels (CHRNA2, CHRNA4, CHRNB2 [all 3 are nicotinic acetylcholine receptors {nAChR} for which carbamazepine has shown the greatest treatment response through selective nAChR inhibition], GABRG2, and KCNT1); (2) genes found upstream of the rapamycin Complex 1 (mTORC1) signal transduction pathway (DEP domain containing Protein 5 [DEPDC5], NPRL2, NPRL3, TSC1, and TSC2); and (3) other genes (CRH, CaBP4, STX1B, and PRIMA1). Epileptic seizures can manifest from these pathogenic variants due to cellular protein dysregulation and neuronal dysfunction (Figure 1) [1].

**FIGURE 1:**
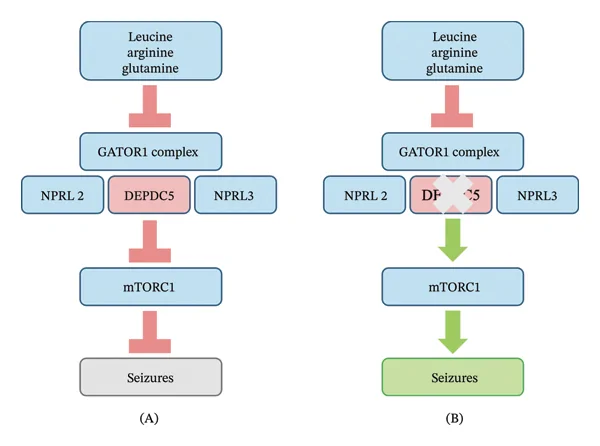
Depiction of a DEP domain containing Protein 5 (DEPDC5) deletion. (A) The normal function of mTORC1 pathway where DEPDC5 is a component of the GATOR1 complex which inhibits mTORC1, thus inhibiting seizures. The availability of enzymes causes inhibition (depicted as an upside‐down red “T”) of the GATOR1 complex which leads to mTORC1 inhibition and, thus seizure inhibition. (B) When DEPDC5 is nonfunctional (indicated by the gray “X”) due to an exon deletion in our patient case; this leads to mTORC1 hyperactivity (indicated by the downward facing green arrow) and, thus seizures. This figure is author‐generated.

Of the pathogenic genes of ADSHE listed above, pathogenic variants in *DEPDC5* and other *GATOR1* complex genes are the most prevalent. These mutations can result in inherited cortical structural abnormalities, increased risk for drug resistant epilepsy, and sleep‐related and diurnal seizures [1]. Most individuals (60%) with DEPDC5‐related epilepsy have normal MRIs [3]. Not all carriers of pathogenic DEPDC5 variants develop focal epilepsy or cortical dysplasia, suggesting incomplete penetrance of pathogenic DEPDC5 variants [1].

Here, we present the case of a father and daughter who both possess a novel DEPDC5 gene deletion on Exon 28. This deletion along with clinical features and diagnostic study results lead to a diagnosis of ADSHE in both patients; ADSHE is managed by lacosamide and brivaracetam in the father and lacosamide alone in the daughter.

## 2. Case Report

This case report describes a father–daughter pair with ADSHE secondary to *DEPDC5* gene deletion in Exon 28. The daughter was 18 years old at presentation and began having sleep‐related seizures at age 12. Her first seizure was unwitnessed and associated with sleep‐related enuresis and tongue biting during sleep followed by postictal headache. Subsequent witnessed seizures were from sleep and characterized by gurgling noises, arousal from sleep, blank stare with eyes open, unresponsiveness to verbal stimuli, and intermittent oral automatisms. These sleep‐related seizures typically lasted two to 3 minutes and were associated with tongue bite but inconsistently associated with urinary incontinence; they were not associated with any extremity jerking or motor movements. Postictally, she would go back to sleep and have no recollection of the event in the morning. Her routine EEG was reported normal; CT head was also unremarkable. Her pediatric neurologist recommended epilepsy monitoring unit admission to characterize her sleep‐related events with differential of parasomnia versus seizures, but family decided not to complete this test. She was not trialed on any antiseizure medications (ASMs) and was lost to follow up from age 14 to 18.

On presentation to adult neurology at age 18, she reported sleep‐related seizures occurring once every 1‐2 months. The semiology of her events is similar to those in childhood and is now characterized by waking from sleep, motionless stare with eyes wide open, hyperkinetic behavior with grunting/guttural noise, stiffening of limbs, heavy breathing, jaw clenching, and drooling associated with tongue bite and urinary incontinence. Sleep‐related seizures last 1‐2 min followed by confusion and inability to follow commands. She tends to wake up a few hours later with a postictal headache. Due to a family history of seizures with similar semiology and suspicion of a frontal lobe focus, autosomal dominant sleep‐related frontal lobe epilepsy was suspected. Lacosamide was titrated to a goal of 100 mg twice daily.

Her brain MRI was ultimately deemed cryptogenic with no clear structural etiology of seizures appreciated. MRI revealed a nonenhancing T2 hyperintense signal of the thalami (right > left) with no evidence of mass lesion, hydrocephalus, and hemorrhage.

The father’s path to an ADSHE diagnosis was more protracted than his daughter’s. He is a 50‐year‐old right‐handed male who had his first seizure at the age of 20; he was treated with phenytoin for one year at that time. He had a second seizure at 22 years of age but remained seizure‐free from age 22 to 47. He experienced a recurrence of both sleep‐related and diurnal seizures at age 47. Levetiracetam was initiated but later discontinued due to severe anxiety. Lacosamide was initiated and progressively titrated up to 250 mg twice daily, and brivaracetam 100 mg twice daily was subsequently added to achieve seizure control.

The father’s MRI revealed an Arnold–Chiari 1 malformation, bilateral hippocampal cysts, coarctation of lateral ventricle frontal horns, and an arachnoid cyst without mass effect or abnormal enhancement. While these findings are intriguing, they were considered incidental with no relevance to his epilepsy diagnosis. He underwent ambulatory video EEG monitoring which captured several typical events characterized by arousal from sleep, sitting up in bed and making grunting/guttural noises lasting 1‐2 min with and without impairment of awareness. Electrographically, the ictal EEG consisted of movement and myogenic artifact with poorly formed epileptiform discharges; this may suggest a deep seizure focus. These episodes occurred multiple times in the early part of the night during both Stage II and Stage III of nonrapid eye movement sleep.

To assist in differentiating between a diagnosis of parasomnia and epilepsy, Frontal Lobe Epilepsy and Parasomnias (FLEP) Scale surveys were completed during each patient’s follow‐up visit. The father received a score of 3 (1 point given for events lasting < 2 min, 1 point for typically having 3–5 events per night, and 1 point for the patient having lucid recall of the events), and the daughter received a FLEP score of 4 (1 point for the events occurring within 30 min of sleep onset, 1 point for having a history of dystonic posturing, tonic limb extension or cramping during events, and 1 point for having highly stereotyped events). Both patients’ scores suggested that nighttime episodes were more consistent with seizures (score threshold ≥ 1) than parasomnias (score threshold ≤ 0).

Subsequently, the father and daughter both underwent genetic testing performed by Labcorp Genetics, Inc. Genomic DNA was extracted from the clinical sample, enriched for targeted regions using a hybridization‐based protocol, and sequenced using Illumina technology. Sequence analysis and deletion/duplication testing of 320 genes associated with genetic epilepsy disorders were performed, combining the Invitae Epilepsy Panel and the Add‐on Preliminary‐Evidence Genes for Epilepsy. All targeted regions were sequenced with a ≥ 50x depth of coverage and aligned to a reference sequence (GRCh37).

Genetic testing identified a novel, heterozygous deletion of Exon 28 in the *DEPDC5* gene (NM_001242896.1) in each patient. The precise genomic breakpoints reside within unsequenced intronic regions where the exact c. coordinates are unknown. This copy number variant was detected using a validated in‐house algorithm specific to exonic deletion and duplication. The deletion spans the following nucleotides (including the flanking nucleotides from Exons 27 and 29 required to complete the reading frame): 5′ggccttgtgtcccgaaaccgccctgaggaggaggaccagtattggctgagtatgggcagaacgttccacaaagtgacgctgaaggataagatgatcacagtgacgcgataccttcccaag3’ causing deletion of the following amino acids: GLVSRNRPEEEDQYWLSMGRTFHKVTLKDKMITVTRYLPK and subjecting the rest of the protein to nonsense‐mediated mRNA decay. The variant has not previously been reported in the literature in those with DEPDC5‐related conditions (Figure 2).

**FIGURE 2:**
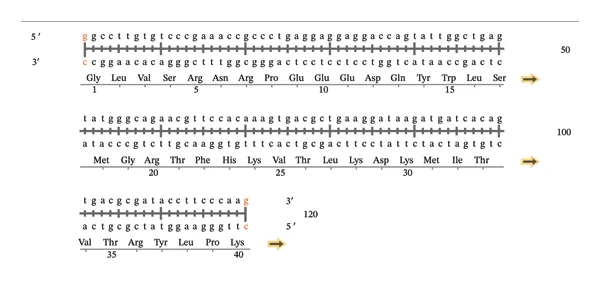
Exon 28 sequence of Chromosome 22. This figure displays the Exon‐28 sequence with the nucleotides in red representing Exons 27 and 29. Below is the translation of amino acids that are deleted along with the Exon‐28 deletion with the remaining protein being out‐of‐frame and, thus have the potential to be nonsense‐mediated mRNA decay. *Image was created with* SnapGene software (from Dotmatics; available at http://snapgene.com).

Many additional family members underwent genetic testing, including the father’s two other daughters. One of his other daughters did have a DEPDC5 deletion; however, she did not have the ADSHE phenotype (Figure 3).

**FIGURE 3:**
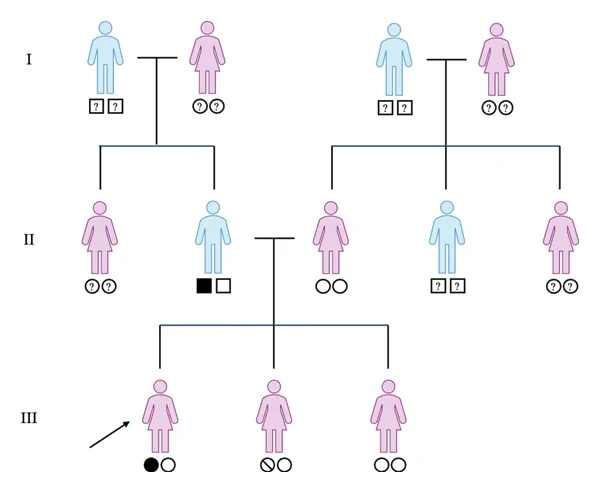
Family pedigree of ADSHE. Men are indicated in blue with 2 squares below, and women are indicated in purple with 2 circles below. White shapes indicate that the patient has neither the genotype nor phenotype; black shapes indicate that the patient has the genotype and phenotype, and a line through the shape means the patient has the genotype but not phenotype. Those who were not genetically tested have a “?” in the shape. The proband is indicated with an arrow. Both the daughter and her father have a heterozygous DEPDC5 deletion which is indicated by a black circle or square. This figure is author‐generated.

Additionally, both the daughter and father were found to have variants of unknown significance (VUS). The daughter had a *SCN5A* variation (associated with heart conditions) and the father had *RHOBTB2* and *SGCE* variations (associated with intellectual disability, dystonia, and infantile onset seizures). However, considering these clinical features were absent, additional workup was not indicated in either patient [4–6].

The pathogenic DEPDC5 deletion of Exon 28 in both father and daughter produces a nonfunctional protein that causes mTORC1 hyperactivity and resultant seizures as discussed above. The father’s and daughter’s individual VUSs were included here to provide context but may not be phenotypically relevant.

The father was initiated on levetiracetam initially but experienced severe anxiety and was switched to lacosamide; brivaracetam was later added for additional seizure control. The daughter remains well controlled on lacosamide, and the father remains well controlled on lacosamide and brivaracetam. These medications were prescribed to the patients prior to their genetic testing, and since the patients’ seizures were well controlled on these medications, they were not changed.

## 3. Discussion

Distinguishing SHE from nonepileptic pathogenic or physiologic sleep behaviors can be clinically challenging. Diagnosis of ADSHE is multifactorial and includes seizure semiology, family history, and neuroimaging/EEG findings [3]. Our patients presented with hypermotor sleep‐related seizures, strong family history of seizures, normal routine EEG findings, and MRIs without epileptogenic foci. Diagnosis of ADSHE for these patients was based on a combination of genetic test results, clinical history, and FLEP scores.

Although the prevalence of ADSHE is unknown, ADSHE itself has been identified in over 100 families worldwide [7]. In one Italian study that looked at 103 patients with SHE, DEPDC5 had the highest number of pathogenic variants (3.9%), compared to other causal pathogenic variants of SHE [2]. This disorder is autosomal dominant with both biallelic and postzygotic pathogenic DEPDC5 variants described in the literature [3]. In this case, both father and daughter were found to have DEPDC5 deletions in Exon 28.

Pathogenic variants of DEPDC5 commonly result in loss of function, impairing the inhibitory activity of the GATOR1 complex on mTORC1 signaling, which may contribute to increased neuronal excitability [8]. Intragenic deletions represent a less frequently identified variant class among pathogenic variants. Bonaglia et al. describe a large deletion from Exons 28 to 38 of DEPDC5 in a child with focal epilepsy, which preserved the reading frame but was predicted to produce a shortened protein lacking the DEP domain [9]. Recently, Zhou et al. reported a splice‐site variant (c.2633+2T > *C*) associated with aberrant splicing of Exon 28 and premature termination codons [10]. In contrast, we report an isolated deletion of Exon 28, predicted to introduce a frameshift and premature termination codon, resulting in nonsense‐mediated decay. This is consistent with the haploinsufficiency mechanism underlying most casues of DEPDC5‐related epilepsy [11]. While previous reports have associated alterations in Exon 28 with focal epilepsy, we identify a pathogenic deletion in this family with ADSHE.

First utilized in 2006, the FLEP scale is a means of distinguishing parasomnias from nocturnal frontal lobe epilepsy based on different clinical features including age at onset, duration, clustering, timing, symptoms, stereotypy, recall, and vocalizations. Positive scores (≥ 1) increase the threshold of suspicion for epilepsy, while negative scores (≤ 0) increase the threshold of suspicion for parasomnias. In our case, the father and daughter both had positive FLEP scores (3 and 4, respectively), indicating an epilepsy rather than a parasomnia diagnosis [12].

Clinical diagnostics for pathogenic variants of DEPDC5 also include MRI. Possible MRI manifestations include focal cortical Dysplasia IIa (cortical dyslamination with dysmorphic neurons), hemimegaloencephaly and rarely polymicrogyria, pachygyria, heterotopia, and corpus callosum hypoplasia [3]. The daughter’s MRI demonstrated subtle nonenhancing thalamic hyperintensity, more pronounced on the right, but no other intracranial abnormalities. The father’s MRI revealed a Chiari 1 malformation, arachnoid cyst, bilateral hippocampal cysts, and coarctation of lateral ventricle frontal horns.

The FLEP Scale is useful for differentiating between sleep‐related seizures and a parasomnia diagnosis, and MRI is useful for identifying structural brain changes that could drive epilepsy; nevertheless, video‐EEG capturing sleep‐related seizure activity, epileptic discharges, or interictal epileptiform abnormalities is the gold standard for SHE diagnosis. However, SHE diagnosis cannot be excluded even if the captured events lack ictal or interictal correlates during wakefulness or sleep due to the potential for a deep‐seated epileptogenic focus. Furthermore, while the patients in these cases likely have a frontal lobe seizure etiology based on the semiology presented, it is possible for SHE to have extrafrontal foci [13, 14]. Routine EEGs for both father and daughter demonstrated a normal background during awake, drowsy, and sleep periods.

Notably, not all carriers of heterozygous pathogenic DEPDC5 variants develop epilepsy, and those who do present with a variety of phenotypes, including familial focal epilepsy with variable foci, ADSHE, familial temporal lobe epilepsy, infantile spasms, and sudden unexpected death in epilepsy [3]. This incomplete penetrance was observed within our family as well, as another daughter carried the same heterozygous Exon 28 deletion without demonstrating the reported phenotype. Our patients both had ADSHE, which is characterized by clustered sleep‐related motor seizures lasting a few seconds to a few minutes. Presentations vary within families and across the broader ADSHE population, from simple arousals to dramatic hyperkinetic tonic or dystonic events. Focal seizures with preserved consciousness are common. The mean age of ADSHE onset is 5 years old, with 80% of individuals developing ADSHE within the first 2 decades of life [3]. However, the age of onset must be between 2 months and 64 years old to meet diagnostic criteria [15]. Over time, ADSHE seizures can become milder and less frequent. Neurological examinations are often normal with preserved cognitive function [3].

ASM treatment response is variable. In this case, the daughter manages her epilepsy with lacosamide, while her father manages his epilepsy with lacosamide and brivaracetam. These patients were already well‐managed on these ASMs prior to genetic testing. Carbamazepine is considered first‐line for ADSHE because it yields 70% remission; it works by selectively inhibiting nAChR, the receptor affected by pathogenic neuronal ligand (CHRNA2, CHRNA4, and CHRNB2) variants. Since these pathogenic variants were not present in these patients, theoretically, a trial of carbamazepine in patients with pathogenic DEPDC5 variants might not yield as high of a treatment success rate compared to patients with pathogenic CHRNA2, CHRNA4, or CHRNB2 variants given the disparate pathophysiology for developing ADSHE.

Thirty percent of patients with ADSHE have refractory epilepsy for which comprehensive surgical evaluation should be explored. While resective surgery could be performed in patients presenting with focal cortical dysplasia, disconnective surgery could be performed in patients presenting with hemimegaloencephaly [16, 17]. In patients without structural brain abnormalities, neuromodulation, such as vagal nerve stimulation, can be pursued [3, 15]. In this situation, both the father and daughter were well controlled with ASMs, and surgical work‐up and treatment were not pursued.

Overall, our case report highlights the possible clinical presentations of ADSHE secondary to pathogenic DEPDC5 variants. Diagnosis involves a combination of genetic testing, EEG, MRI, and FLEP scores as appropriate. Further research is needed to determine optimal treatments for ADSHE caused by pathogenic DEPDC5 variants.

## Author Contributions

Conceptualization: Elisabeth M. Benham and Chetan S. Nayak; methodology: Julianna L. Barbaro, Elisabeth M. Benham, Ayushi Ambekar, and Chetan S. Nayak; validation: Julianna L. Barbaro, Elisabeth M. Benham, Ayushi Ambekar, and Chetan S. Nayak; formal analysis: Julianna L. Barbaro, Elisabeth M. Benham, Ayushi Ambekar, and Chetan S. Nayak; investigation: Julianna L. Barbaro, Elisabeth M. Benham, Ayushi Ambekar, and Chetan S. Nayak.; data curation: Julianna L. Barbaro, Elisabeth M. Benham, and Chetan S. Nayak; writing–original draft preparation: Julianna L. Barbaro; writing–review and editing: Julianna L. Barbaro, Elisabeth M. Benham, Ayushi Ambekar, and Chetan S. Nayak; visualization: Julianna L. Barbaro, Elisabeth M. Benham, Ayushi Ambekar, and Chetan S. Nayak; supervision: Elisabeth M. Benham and Chetan S. Nayak.

## Funding

The authors do not have any financial disclosures and did not receive any funding for completion of this case report.

## Disclosure

All authors have read and agreed to the published version of the manuscript. The authors confirm that we have read the Journal’s position on issues involved in ethical publication and affirm that this report is consistent with those guidelines.

## Ethics Statement

All work in this manuscript was conducted in accordance with the Declaration of Helsinki (1964).

## Consent

Written informed consent was obtained from both patients in this case report for publication of this case report and accompanying images and genetic test results in accordance with the International Committee of Medical Journal Editors (ICMJE). This case report was exempt from IRB review.

## Conflicts of Interest

The authors declare no conflicts of interest.

## Data Availability

The data supporting the findings of this case report are available from the corresponding author upon reasonable request and may be made available to the journal for editorial review.
